# Findings From Three Neurodevelopmental Psychiatry Educational Events Aimed at Medical Students and Junior Doctors

**DOI:** 10.1192/bjo.2022.154

**Published:** 2022-06-20

**Authors:** Nasreen Shaikh, Niraj Singh, Mary Barrett, Samuel Tromans, Prabhleen Singh Jaggi

**Affiliations:** 1 Nottinghamshire Healthcare NHS Foundation Trust, Nottingham, United Kingdom; 2 Leicester Partnership NHS Trust, Leicester, United Kingdom

## Abstract

**Aims:**

To review feedback from three Neurodevelopmental Psychiatry educational events attended by medical students and junior doctors, to establish their impact and whether they can influence interest in Psychiatry/Neurodevelopmental Psychiatry as a career.

**Methods:**

Three events were organised to a) increase understanding of Neurodevelopmental Psychiatry and b) promote career interest in the specialty, aiding recruitment efforts. Two were Face to Face Events (FFEs) whereas one was an Online Event (OE) in keeping with COVID-19 restrictions.

The programme for the events was varied including key clinical topics such as Intellectual Disability, autism, ADHD and epilepsy as well as leadership, management, research and training information. Presentations were approximately 20 min in duration. 31 delegates attended the 2018 FFE, 28 attended the 2019 FFE and 65 attended the 2020 OE.

The 2018 FFE and 2020 OE were primarily attended by medical students whereas the 2019 FFE was attended primarily by junior doctors.

Delegates rated each presentation from 1 (poor) to 5(excellent) and provided comments. At the 2018 and 2019 FFEs we assessed impact on career interest.

**Results:**

- The majority of delegates from both FFEs agreed that such events helped to facilitate understanding of neurodevelopmental psychiatry and encourage recruitment to psychiatry.
- The majority of delegates at the 2019 FFE agreed that their interest in a career in neurodevelopmental psychiatry had increased following attendance
- Attendance was highest at the 2020 OE and overall rating was 4.63/5.
- Across the events, popular topics were Autism, Career path and Physical Health needs in Intellectual disability.
- Themes in terms of comments included “friendly, inspiring speakers” and “opportunity for interactivity” (noted at OE).

**Conclusion:**

Both the OE and FFEs were enjoyed by medical students and junior doctors.

Analysis showed key topics such as autism attract interest but also that diverse topics in different formats are important. Human factors that seemed important included inspiring, friendly speakers and a relaxed, interactive atmosphere. OEs are cost-effective and have the potential to attract a bigger audience but may present a challenge in terms of interaction. FFEs impact positively on career interest and this needs to be assessed further in terms of online events.

